# Malaria prevalence, knowledge, attitude, and practice among febrile patients attending Chagni health center, Northwest Ethiopia: a cross-sectional study

**DOI:** 10.1186/s40794-021-00146-2

**Published:** 2021-07-05

**Authors:** Bogale Belay, Tegenu Gelana, Araya Gebresilassie

**Affiliations:** Department of Zoological Sciences, Addis Ababa University, Addis Ababa, Ethiopia

**Keywords:** Chagni, Febrile illness, KAP, Malaria, Prevalence

## Abstract

**Background:**

Ethiopia has achieved considerable progresses in the prevention and control of malaria in the past decades; hitherto it is a formidable health concern and socio-economic impediment. This study aimed at assessing the magnitude, knowledge, attitudes and practices towards malaria among febrile patients attending Chagni health center, northwest Ethiopia.

**Methods:**

Health facility-based cross-sectional study was conducted to estimate the prevalence of malaria and KAP towards malaria among febrile patients at Chagni health center in Chagni Town during September 2017 to February 2018. In order to determine the magnitude of malaria, finger prick blood samples were collected and thick and thin smears were prepared and microscopically examined for the presence of malaria parasites. A pre-tested structured questionnaire was also applied to assess KAP of suspected malaria patients, attending the health center. Data were analyzed using SPSS version 20.0.

**Results:**

Prevalence of malaria among febrile patients, who visited the sampled health facility, was 7.3%. Of these, *Plasmodium falciparum*, *P. vivax*, and mixed infections accounted for 55, 44.3 and 0.7% of the cases, respectively. This study also revealed that 97% of the respondents had ever heard about malaria and recognized it as a serious health problem. Mosquito bite was identified as the main malaria transmission. Taking drug (86.3%), use of mosquito nets (73.3%), drain stagnated water (68%), and house spay with insecticides (66%) were mentioned as the main malaria prevention methods. Mosquito net coverage and utilizations in the prior night were 98 and 75%, respectively. Indoor residual spraying (IRS) coverage was 99%, of which 77.5% of study participants’ houses have been sprayed in the last 6 months.

**Conclusions:**

The current study revealed that prevalence of malaria among febrile illnesses in the study area was relatively low (7.3%) with a high proportion of *P. falciparum*. Besides, participants had adequate knowledge, encouraging attitudes, and good practices about prevention and control of malaria. However, some misconceptions on malaria disease, its transmission, and prevention have been noted that actually require due attention by the concerned stakeholders. The findings of this study could be used as important inputs for the implementation of effective malaria prevention and control methods, including community health education programs, and scaling up coverage of evidence-based interventions.

**Supplementary Information:**

The online version contains supplementary material available at 10.1186/s40794-021-00146-2.

## Background

Malaria is a leading public health problem in Ethiopia where an estimated 68% of the population lives in malarious areas and three quarters of the total land mass is regarded as malarious [1]. Malaria has been one of the main causes of hospitalization and deaths in the country. In 2009/2010, it was the most important cause of outpatient visits and health facility admissions, accounting for 14% of outpatient visits and 9% of admissions in the country in 2009/2010 [2]. *Plasmodium falciparum* and *P. vivax*, which are distributed all over the endemic regions of the country, account for about 60 and 40% of malaria cases, respectively [3].

Varying topographical and climatic features contribute to the seasonal and unstable malaria transmission pattern in Ethiopia, which is usually characterized by frequent focal, cyclic, and widespread epidemics [3]. In general, the peak of malaria incidence follows the main rainfall season in July, August and September each year [3]. Previous cross-sectional studies of malaria prevalence in Ethiopia have demonstrated the wide range (2.8 to 39.6%) of malaria prevalence [4–8].

Malaria is one of the major public health problems in Amhara Regional State. In 2012, a total of 1,127,241 cases of malaria were reported in this Region, out of a population of 19,867,817 habitants [9]. West Amhara, constituting five administrative zones accounted for 93.1% of the total malaria burden, of which West Gojjam had the greatest number of cases (404,926) [6]. In Awi zone, malaria counted for 259,009 outpatients, 43,131 in-patients, and 1 death from 2011 to 2020 [10]. Chagni town, which is located in this zone, is among the major malarious areas in the region requiring particular attention.

In the past decade, considerable scale-up and implementation of key malaria interventions, including insecticide-treated nets (ITNs), indoor residual spraying of households with insecticide (IRS), and rapid diagnostic tests (RDTs) of malaria coupled with prompt and effective case management with artemisinin-based combination therapy (ACT) resulted in a significant reduction in the prevalence of the malarial in Ethiopia [11, 12]. However, such achievements have been faced with many challenges, including insecticide resistance of vectors, drug resistance of parasites, under-utilization of intervention, increased number of man-made mosquito breeding sites; gaps in service delivery and health system weaknesses [3, 13–15]. Moreover, poor community awareness toward malaria and control intervention practices have also been mentioned [16].

Studies involving community knowledge, attitudes and practices (KAP) have also been carried out in Ethiopia regarding the level of awareness about malaria and associated risk factors to its transmission and control measures [13, 17–20]. Such reports evidenced the presence of misconceptions about malaria causation, symptom identification, and treatment of malaria among communities, in particular within rural areas. In addition, practices for the control of the disease are inadequate, suggesting the need for health education to raise the community’s awareness about the disease. Considering these issues, fostering community KAP that help to gauge the implementation and effectiveness of proven malaria interventions in a given locality is vital to ensure scale-up of control options to sustainably prevent and control the disease.

The aim of the present study was therefore to determine the current prevalence of malaria infection among febrile patients attending Chagni health center, northwest Ethiopia from September 2017 to February 2018. In addition, the study focused on assessing patient’s knowledge, attitude, and practice of malaria and its control.

## Methods

### Study area

The study took place in Chagni Town in Amhara Regional State, northwest Ethiopia. Situated in the Awi Zone, the town has a longitude and latitude of 10°57′N 36°30′E, and an elevation of 1583 m above sea level. The town is located 505 km from Addis Ababa. The mean annual temperature and rainfall in the town are 21.6 °C and 1896.6 mill liters, respectively (Ethiopian Meteorology Agency, unpublished data). The town is divided into five *kebeles* (lowest administrative centers). The town has a total population of 39,292 of whom 19,165 were men and 20,127 women [21]. Two public health institutions (one hospital and one health center) and four medium private clinics are found in the town that provides healthcare service to the society.

### Study design

The study followed facility-based cross-sectional design between September 2017 and February 2018.

### Inclusion and exclusion criteria

For the prospective malaria prevalence study, malaria suspected individuals who had complains of febrile illness in the health center from September 2017 to February 2018 were included. On the other hand, patients who did not give consent and assent, as well as had been in the Kebeles for less than 6 months were excluded from the study.

For KAP study, febrile patients who were > 18 years and permanent residents in the five kebeles of the town were included. Alternatively, study participants who cannot communicate due to impairment or sever sickness and mentally sick people, those people who did not provide consent and assent, were less than 18 years, and had been in the kebeles for less than 6 months, were excluded.

### Sample size and sampling techniques

All of the 4077 malaria suspected patients registered in the health centre from September 2017 to February 2018 were the sample sizes to determine the current prevalence of malaria. In the meantime, the sample size for assessing the KAP of participants was calculated using a single population proportion formula (*n* = z^2^p(1-p)/d2 [22]; where *n* = the sample size, z = 1.96 at 95% confidence interval (CI), d = margin of error at 5%, *P* = 0.78 (78% proportion of population who had good knowledge towards malaria and its prevention was taken from a study done in Amhara region [1]. After adding 5% non-response rate, the final sample size became 274.

For KAP study, a systematic simple random sampling method was used to enroll the study participants. First, the proposed sample size was proportionately allocated to each kebele. Thereafter, individual participants from each kebele who came to health centre were systematically selected by taking every third attendant from random start on the health centre up to the sample size of the population is reached.

### Data collection

#### Blood film collection and testing

Data was collected from September 2017 to February 2018. Blood slide samples were taken from compliance of febrile patients at Chagni health centre. Blood film collection was carried out by experienced medical laboratory technicians by pricking the finger with disposable blood lancet. Throughout the data collection, microscopy was used for the detection and species identification of *Plasmodium* parasites by examination of peripheral smears of stained blood films, as per the WHO protocol. Thick and thin blood smears were taken and identification numbers marked on the thin films. The thin films fixed using 100% methanol and all slides were stained with 3% Giemsa solution for 20 min. The staining technique and blood film examination was conducted according to a standard of WHO protocols. Then, parasite positivity was determined from thick smear and species identification was carried out from thin smear slide preparation. Malaria slides were stained with Giemsa and examined under 100x microscopes for the presence of malaria parasites. Parameters such as date of examination, total clinically treated and confirmed cases in months, types of malaria species, and socio-demographic data such as age and sex were collected of patients were collected on patient registration book. All individuals who had fever on physical examination and were positive for malaria parasites during blood film examination were offered anti-malarial treatment as per national guidelines [4].

#### Measurement of KAP

In order to measure KAP of the study participants about malaria and its prevention and control practices, a structured questionnaire was designed and applied. The questionnaire covered socio-demographic details, knowledge about malaria transmission, treatment and prevention, and risk perception of the disease. It also covered bed net ownership and use, prevention and treatment practices of the respondents. The questionnaire was first developed in English and translated into Amharic (the local language), and then pre-tested in non-selected patients for assessing content validity, appropriateness, and question comprehensibility. It was administered to 274 systematically randomized study participants selected from febrile patients, visiting the health center during September 2017 to February 2018. Two trained laboratory technicians from the health center were selected to collect data. Training was given to the data collectors on how to conduct the interview, content of the questionnaire, data quality, and ways to approach respondents. Data were checked for completeness, and incomplete questionnaires were returned to data collectors for correction by revisiting the concerned interview.

#### Data analysis

Data were checked for completeness and consistency, entered in to Microsoft Excel data sheets, and exported in to Statistical Package for Social Science (SPSS) version 20 software for data analysis. Descriptive statistics were carried out to measure relative frequencies, percentages, averages, and relative frequencies of the variables. The chi-squared test was used for prospective malaria prevalence data to determine differences between age, sex and seasons and malaria parasite distribution. Statistical significance was defined at *P < 0.05*. The analyzed data was presented using tables and figures.

## Results

### Malaria prevalence at health facility survey

In this study, a total of 4077 malaria suspected patients were microscopically diagnosed at Chagni Health Center from September 2017 to February 2018 (Table 1). Of these, 296 (7.3%) were slide positive for malaria with mean monthly malaria cases of 49.3. The mean monthly slide positivity rate was 6.95%, though the number of malaria suspected patients who visited the health center showed fluctuating trend each month. Malaria case distribution by months had significant fluctuating trend (χ^2^ = 14.194, df = 5, *P <* 0.05; Table 1). Transmission of malaria from September to November was higher, 194 (65.5%) whilst the dry season of the year from December to February had lower case, 102 (34.5%).

**Table 1:** Monthly profile of malaria positive patients at Chagni health center (September 2017–February 2018)

Month	Examined number	Confirmed. No (%)	*P-Value*
September	718	37 (5.2)	0.004*
October	986	97 (9.8)	
November	773	60 (7.8)	
December	662	46 (6.9)	
January	547	32 (5.9)	
February	391	24 (6.1)	
Total	4077	296 (7.3)	

*Statistically significant at *P <* 0.05 (χ2 = 14.194, df = 5, *P* = 0.004)

### *Plasmodium* parasite distribution with socio-demographic variables

*Plasmodium falciparum* and *P. vivax* were the only species in the study area, where *P. falciparum* accounted for 163 (55%), *P. vivax* was 131 (44.3%), and the rest 2 (0.7%) were mixed infections of both species. Of the total patients examined, 2103 were males and 1974 were females (Table 2). The infection rates among males were 164 (55.4%) and females were 132 (44.6%), albeit this association of malaria cases with sex was not statistically significant (χ^2^ = 1.868, df = 1, *P* > 0.05).

**Table 2:** Distribution of malaria cases by sex at Chagni health centre from September 2017-Feburary 2018

Sex	Total cases examined	Slide positive No. (%)	*P. falciparum* No. (%)	*P. vivax* No. (%)	Mixed infection No. (%)	*P-value*
Male	2103	164 (55.4)	89 (54.3)	74 (45.1)	1 (0.6)	0.172
Female	1974	132 (44.6)	74 (56)	57 (43.2)	1 (0.8)	
Total	4077	296 (100)	163 (55)	131 (44.3)	2 (0.7)	

**Fig. 1:**
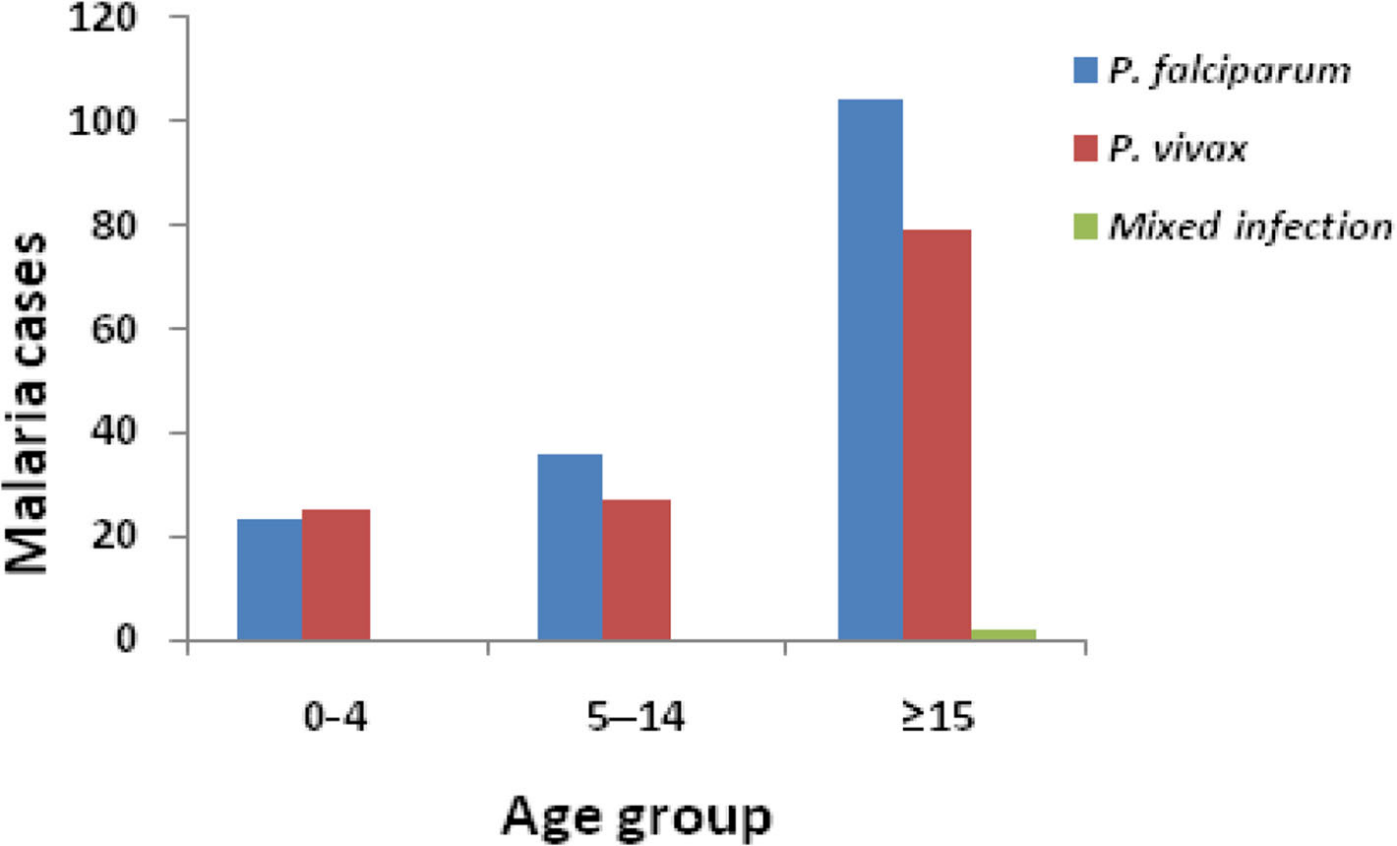
Prevalence of *Plasmodium* spp. in different age groups in Chagni Health Centre from September 2017-Feburary 2018

The distribution of parasite species in relation to age is presented in Fig. 1. Distribution of malaria varied among different age groups, ranging from 4.7 to 8.5% (*χ*^*2*^ *= 14.448, d.f. = 2, P = 0.001*). Adults (≥15 years) were the most affected group 185 (8.5%), followed by 5–14 and under 5 age categories, with prevalence rates 63 (7.1%) and 48 (4.7%), respectively. *P. falciparum* species proportion of positive cases were higher in 5–14 age group, followed by ≥15 years, and under 5 age group with a prevalence rate of 57.1% (36/63), 56.2% (104/185) and 47.9% (23/48), respectively (Fig. 1). Similarly, the age group ≥15 was more affected by *P. vivax,* 79 (42.7%) compared to 5–14, 27 (30.7%) and below 5 year, 25 (52.1%) age categories. There were two mixed cases detected in both male and female individuals of age groups ≥15 years of age.

### Socio-demographic characteristics for KAP survey

We enrolled a total of 274 individuals with a 100% response rate (Table 3). Out of the total respondents, 154 (56.2%) and 120 (43.8%) were males and females, respectively. The age of the respondents ranged from 18 to 74 years, with the median age of 32.4 years. Most of study participants 128 (46.7%) were married. The majority of respondents [118 (43.1%)] had no formal education. The average family size of the respondents was 3.84. The main economic activities by study respondents were daily labor 95 (34.7%), government employment 64 (23.4%), trading 48 (17.5%), agriculture 33 (12%), and other 34 (12.4%) (Table 3).

**Table 3:** Socio-demographic characteristics of respondents Chagni town, 2018 (*n* = 274)

Variable	Category	Frequency (%)
Sex	Male	154 (56.2)
	Female	120 (43.8)
Age	18–24	90 (32.8)
	25–34	73 (26.6)
	≥35	111 (40.5)
Marital status	Single	86 (31.4)
	Married	128 (46.7)
	Divorced	14 (5.1)
	Widowed	46 (16.8)
Family size/HH	1–4	120 (43.8)
	5–7	115 (42)
	8 and above	39 (14.2)
Educational status	Illiterate	118 (43.1)
	Read and write	96 (35)
	Primary school	29 (10.6)
	Secondary school and above	31 (11.3)
Occupation	Civil servant	64 (23.4)
	Farmer	33 (12)
	Merchant	48 (17.5)
	Daily laborer	95 (34.7)
	Other	34 (12.4)
Family income	< 500	46 (16.8)
	500–1000	121 (44.2)
	> 1000	107 (39)
Religion	Orthodox	183 (66.8)
	Muslim	78 (28.5)
	Protestant	9 (3.2)
	Catholic	4 (1.5)
Mass media	Radio	24 (8.8)
	Television	213 (77.7)
	No	37 (13.5)

*HH* household

### Knowledge and attitude of respondents towards malaria and mosquitoes

Malaria related knowledge of the participants is summarized in Table 4. The majority of the respondents [266 (97%)] had ever heard about malaria and the same number of respondents believed that malaria is one of serious and life-threatening diseases of the community. The most common sources of malaria related information were mass media (radio and television) 229 (86.1%), and health facility education 37 (13.9%). One hundred sixty two (60.9%) of the respondents mentioned that malaria is transmittable from person to person, of whom 156 (96.3%) and 6 (3.7%) associated the transmission with the bite of mosquitoes and drinking contaminated water, respectively (Table 4).

**Table 4:** Knowledge of respondents related to symptoms and transmission of malaria, and mosquito behaviors, Chagni town, 2018

Variables	Category	Frequency (%)
Ever heard about malaria	Yes	266 (97)
	No	8 (3)
Malaria a health problem	Yes	266 (97)
	No	8 (3)
Source of information	Health facility	37 (14)
	Mass media (Radio and TV)	229 (86)
Malaria transmissible	Yes	162 (60.9)
	No	104 (39.1)
Ways of malaria transmission	Through mosquito bite	156 (96.3)
	Drinking contaminated water	6 (3.7)
Signs/symptoms of malaria	Fever	258 (97)
	Chills and shivering	257 (96.6)
	Headache	254 (95.4)
	Loss of appetite	248 (93.2)
	Joint pain	242 (90.9)
	vomiting	235 (88.3)
When mosquitoes bite mostly	Day	6 (2.3)
	Night	245 (92.1)
	Anytime	5 (1.9)
	Don’t know	10 (3.8)
Mosquito breeding sites	Stagnant water	228 (85.7)
	Waste material	25 (9.4)
	Houses	8 (3)
	Don’t know	5 (1.9)

Percentages do not add up to 100 because of multiple responses

Fever, chills and shivering, and headache were the most frequently mentioned malaria symptoms reported by 97, 96.6, and 95.4% of the respondents, respectively (Table 4).

Of the respondents, the majority (97%) stated malaria as a preventable and curable disease, while 2.6% considered the opposite and 0.4% did not know. Taking drug 226 (85%), using mosquito nets 192 (72.2%), draining stagnant water 178 (67%), and IRS 173 (65%) were the most commonly mentioned preventive strategies. Most of the respondents 218 (82%) believed that sleeping under mosquito net protect from mosquito bites (Table 5).

**Table 5:** Knowledge and attitude of respondents regarding preventive methods of malaria, Chagni town, 2018

Variables	Category	Frequency (%)
Malaria is preventable and curable	Yes	258 (97)
	No	7 (2.6)
	I don’t know	1 (0.4)
Preventive methods	Drug	226 (85)
	Use of mosquito net	192 (72.2)
	Drain stagnant water	178 (67)
	Indoor residual spray (IRS)	173 (65)
Advantage of mosquito nets	Protect from mosquito bite	218 (82)
	Sleep better	24 (9)
	Protect from the bite of other insects	24 (9)

### Malaria prevention practices

Regarding treatment seeking behavior, 243 (91.4%) of the respondents had sought treatment within the first 24 h of symptom onset while the rest, 23 (8.8%) had delayed treatment seeking behavior (Table 6). Of the respondents, 243 (91.4%) responded that they sought treatment from health center/clinic, 15 (5.6%) while 8 (3%) of them practice self-medication by procuring drugs from drug stores and using traditional medicines such as garlic and leaf of neem tree, respectively.

**Table 6:** Practices of respondents towards malaria prevention and control in Chagni town, 2018

Variable	Category	Frequency (%)
Seek treatment in 24 h of onset of symptoms	Yes	243 (91.4)
	No	23 (8.8)
Seek treatment from	Health center/clinic	243 (91.4)
	Drug shop/Pharmacy	15 (5.6)
	Look for traditional medicine	8 (3)
ITN possessed (*N* = 274)	Yes	268 (98)
ITNs per family	One	20 (8)
	Two	167 (62)
	More than two	81 (30)
ITN used previous night	Yes	201 (75)
	No	67 (25)
Who uses ITN	Husband and wife	44 (22)
	Pregnant mother and children	149 (74)
	All family	8 (4)
Seasonal use of ITNs	All year	201 (75)
	During malaria season	67 (25)
Reason for not using ITN	Do not prevent malaria	24 (36)
	Burning sensation	43 (64)
	Damage (worn-out)	6 (100)
House spray with insecticide (*n* = 274)	Yes	271 (99)
	No	3 (1)
IRS usage	Last 6 months	210 (77.5)
	Last 12 month	61 (22.5)

ITN-Insecticide treated net; IRS-Indoor residual spraying

The majority (98%) of interviewees reported that they possessed at least one insecticide-treated mosquito net (ITN) in their houses. Out of which, 167 (62%), 81 (30%), and 20 (8%) had two, more than two, and one ITN per household, respectively. The majority (75%) of the ITN owners claimed that they slept under a bed net the previous night while the rest of 25% did not. A similar figure (74%) replied that pregnant mother and children were given priority to sleep under bed net, followed by father and mother (22%), and the remaining family members (4%). It was also indicated that three fourth of the individuals who owned ITN reported that they utilize bed net regularly /every night, whilst 25% of respondents stated they use it during the malaria season. Of those who are not currently using bed net, 192 (77%) mentioned lack of access, 20 (8%), and 37 (15%) mentioned other reasons. The reasons for not using bed net were that fear of burning sensation (64%), and 36% lack of awareness (Table 6).

The study also revealed that IRS is one of the most important malaria prevention methods practiced in the locality with the overall coverage of 99%. Out of the respondents, 77.5 and 22.5% of them stated that their house was sprayed with chemicals in the last 6 and 12 months, respectively (Table 6).

## Discussion

In this study, we found that relatively small percentage of febrile patients visiting Chagni health center had microscopically confirmed malaria parasitemia. The overall malaria positivity rate was 7.3%. This figure is comparable with the result of the study done in Kombolcha health facility, north-central Ethiopia that reported a prevalence of 7.52% [23]. Nonetheless, the finding of this study contradicts with previous studies from southern and northern Ethiopia, reported overall malaria positivity rates between 11.5 and 28.1% among patients visited health facilities [5, 24, 25]. Possible factors for observed variations might be differences in the time of studies, microclimate, altitude, community awareness about malaria bed net application, its transmission, and health seeking behavior, and malaria intervention practices.

The dominant *Plasmodium* species detected in the current study participants was *P. falciparum*. This finding is congruent with national figures and other similar studies in parts of Ethiopia that reported preponderance of *P. falciparum* than *P. vivax* [3, 8, 26, 27]. However, this is incongruity with the previous report from Jimma Town, which reported a higher prevalence of *P. vivax* than *P. falciparum* [28]. The reason why *P. falciparum* dominated over *P. vivax* in the study area could be related to drug resistance pattern, and gap of program performance.

In the present study, more males (55.4%) were affected by malaria than females (44.6%). This finding is concurrent with studies from several localities in Ethiopia that reported higher malaria burden among males than females [23, 26, 27]. The higher prevalence rate in males might be due to the fact that males are usually engaged in outdoor activities at dusks and dawns, coinciding with the peak biting hours of the exophagic mosquito species. In addition, males often travel as seasonal migrant laborers to different malarious parts of Ethiopia to perform agricultural activities, thereby exposing them to the higher risk of contracting malaria infection. Conversely, this was not similar with a study conducted in Amhara region where the prevalence of malaria was relatively higher among females (60%) than males (40%) [29].

Regarding the age groups, the burden of malaria morbidity was more concentrated in the adults of age 15 and above. Studies elsewhere in Ethiopia have also shown that the risk of malaria infection varied by age with some reporting more susceptibility to malaria infections among males in the age groups > 15 years [7, 26, 27, 30]. The contributing factors for such higher burden of disease among adults might be due to their frequent engagement in different activities like agriculture, trade and other occupational risks that increase the exposure to infective mosquito bites. Lower cases of malaria in children under 5 years of age was detected, which could be linked to their reduced exposure to infected mosquito bite due to good malaria awareness and control and prevention practices by their guardians.

The results revealed that most of the respondents (97%) had ever heard about malaria and similar number of respondents believed that malaria is one of the serious diseases of the community, which is in line with previous reports in Ethiopia and elsewhere [20, 31, 32]. Study subjects also cited that the most common source of information about malaria was mass media (radio and television) (86.1%), followed by health facility education (13.9%), indicating that these communication channels are essential vehicles to deliver malaria-related information to the community. Elements of IVM such as community malaria education using mass media such as radio and TV have been implemented by the National malaria control program since 2006 [33]. This is similar with those previously reported results from Africa, in which over 90% of individuals in malaria endemic areas are aware of malaria and that mass media (television and radio) and health education by health facilities are the most commonly cited source of malaria information sources [34, 35].

Mosquito bite has been identified as the principal malaria transmission as shown in some studies in Ethiopia and elsewhere in Africa [18, 20, 34–37]. In Ethiopia, regular practice of awareness creation in the communities about health issues through health extension workers and mass media such as radio and television brought remarkable behavioral changes in the control and prevention of communicable diseases [33]. It can be presumed that this factor has contributed to the high level of awareness observed in the study participants regarding the causes and transmission of malaria in the area.

Fever, headache, chills and shivering, loss of appetite and vomiting were mentioned as sign and symptoms of malaria. Similar results were found from different KAP studies in other regions of Ethiopia [18, 20, 36, 38]. Prominently, large majority of subjects linked mosquito biting time during night time and their main breed sites to stagnant water, which is comparable with previous studies in Ethiopia and elsewhere [33, 37, 38]. This correct understanding of mosquito behavior among participants of the present study is encouraging to implement appropriate malaria preventive measures and for the proper utilization of ITNs.

Similar to other studies in Amhara region and other parts of Ethiopia [18–20, 39], great majority (97%) of participants believed that malaria is preventable and curable disease. Taking drug, use of mosquito nets, drain stagnated water (mosquito breeding sites), and house spay with insecticides were the main types of malaria preventive measures frequently reported by the present study participants. This is in line with previous reports from Tanzania [35] and Iran [40].

Around 91.4% of participants go to the nearest health service within the 24 h upon the occurrence of the first malaria symptoms. This was further substantiated by the observation that about 91.4% of participants sought treatment at health facilities, suggestive of a good practice of treatment-seeking behavior at health structures. Generally, treatment seeking behavior for malaria diseases showed improvements across the country in recent reports [1]. However, the commitment among few participants to bring people with fever to health facilities within 24 h since the onset of clinical symptoms remains low, and that they rely on the use of self-administered drugs and traditional medicines, which are common practices in parts of Africa [18, 34, 41]. Treatment seeking behavior is important for early case detection and management so that transmission would be reduced. Therefore, promotion of health education to raise awareness is very crucial to direct the wider community to seek timely treatments at the health structures earlier upon the occurrence of malaria symptoms.

This study also demonstrated that around 98% of participants had least one ITN, of which 75% of them claimed that they slept under a bed net the previous night, which is consistent with previous studies elsewhere in Ethiopia [19, 38, 42]. Yet, quarter of the respondents did not use bed net the previous night. It is strongly envisaged that ITN ownership itself will have little impact on the burden of malaria unless people regularly use it. Factors such as lack of access to ITNs, discomfort due to heat, fear of burning sensation, low level of awareness about its benefit, and lack replacement schemes for worn out ones were cited as the reasons for not using ITNs in this study. Scaling up community awareness through health education has proved to be effective and has effectively alleviated misconceptions on malaria disease, its transmission, and prevention practices [33, 43, 44]. The study further showed that most of the communities give priority for pregnant mother and children to sleep under bed net, which is comparable with results reported from other studies elsewhere in Ethiopia [20, 36].

Our data also showed that IRS is one of the most important malaria prevention methods practiced in the locality with the overall coverage of 99%. Houses of more than 77% of the respondents get sprayed during spraying campaign, result that is comparable with the reports from Jiga area and Shewa Robit district of north-western Ethiopia [6, 18]. This result asserts the demand to expand the coverage and frequency of IRS in malaria endemic areas in order to achieve an already targeted plan of 100% spraying of households before and throughout the transmission for effective prevention and control of malaria [45].

Limitations of the study design and the methods of data collection might create some potential for biases in this study. The cross-sectional design of the study with a focus of recruiting participants who sought healthcare might have influenced their knowledge and practices in malaria prevention and treatment. In addition, data collection relied on information given by the interviewees so that practices such as presence and use of ITN or treatment seeking practices could not be verified.

## Conclusions

In conclusion, the current study illustrated that prevalence of malaria among febrile illnesses in the study area was relatively low (7.3%) with a dominance of *P. falciparum*. It has also been indicated that study subjects had adequate knowledge, encouraging attitudes, and good practices on malaria prevention and control. Nonetheless, some misconceptions on malaria disease, its transmission, and prevention persist as reported in this study, essentially require due attention device effective intervention to improve these misconceptions and lack of [proper information. The findings of this study would make inroads into the implementation effective malaria interventions in the area and beyond focusing on enhancing community awareness and scaling up coverage of evidence-based interventions.

## Supplementary Information

**Additional file 1.** Questionnaire used in KAP Study.

## Data Availability

All data underlying the findings are available from corresponding author on reasonable request. All relevant data are within the manuscript.

## Abbreviations

KAP: knowledge, attitudes and practices
IRS: Indoor residual spray
ITNs: Insecticide-treated nets
SPSS: Statistical Package for the Social Science
WHO: World Health Organization
