# Supplementary material for: Malaria prevalence, knowledge, attitude, and practice among febrile patients attending Chagni health center, Northwest Ethiopia: a cross-sectional study
Source: Trop Dis Travel Med Vaccines. 2021 Jul 5;7:20. doi: 10.1186/s40794-021-00146-2 (PMC8256592; doi:10.1186/s40794-021-00146-2)
Supplement: Supplementary file 1 — Additional file 1. Questionnaire used in KAP Study. [file 40794_2021_146_MOESM1_ESM.docx]

# Additional File 1 - Questionnaire used in KAP Study

Good morning/afternoon. My name is_______________________ and I am Master’s (MSc) student at Addis Ababa University.

Dear respondents,

You are kindly selected to participate in a study titled **“**Malaria prevalence, knowledge, attitude, and practice among febrile patients attending Chagni health centre, northwest Ethiopia: a cross-sectional study”. The purpose of the study is to determine the magnitude of malaria and collect information on knowledge, attitudes and practices of the study population towards malaria and its control for the development of a strategy in order to minimize possible negative impacts and maximize the positive ones with this action.

Your participation is voluntary and anonymous, and you can decide to answer any or all of the questions. Your name and any information you provide will be held in strictest confidence and will not appear on any documents or publications.

Do you want to ask about the research?

Can I start the questions?

Thank you in advance for agreeing to participate in the study.

Date (DD-MMM-YYYY): ___________________

Name of district_________________________ Kebele name____________________

Participant Identification number _____________________________

1. **Socio-demographic characteristics of Household**
2. Sex A. Male B. Female
3. Age (Years) _________
4. Where is your permanent residence? A. Rural B. Urban
5. How long you lived in the current location? (in years) ________________________
6. Current marital status A. Single B. Married C. Divorced D. Widowed
7. Family size ( number)__________
8. Educational status A. Illiterate B. Read &write C. Primary school D. Secondary school and above
9. What is your major current occupation? (Whatever you do to earn money)?
10. Employed
11. Businessman/women
12. Farmer
13. Housewife
14. Daily labourer
15. Other specify ___________
16. What is your religion? A. Orthodox B. Muslim C. Protestant D. Catholic E. Other__
17. What is the monthly income (on average) of your household including your own? Please, specify___________________
18. Do you or any member your family have any of the following media?

A. Television B. Radio C. Telephone D. Books or magazine E. No F. Other specify____________

1. **Housing type and its environmental factors**
2. Housing type: ________________________
3. How many rooms have your home? **_____________**
4. Floor type A. Soil-earth B. Cemented
5. Type of roof A. Corrugated iron sheets B. Thatched C. Earthen roof D. Others ________
6. Main material of the room’s wall? A. Mud blocks B. Sticks C. Corrugated metal D. sticks and mud E. Other specify_____
7. Wall characteristics A. Cracks inside walls of house B. Cracks outside walls of house C. Uncracked interior walls D. Uncracked exterior walls
8. Is eave/window present? A. Yes B. No
9. Any cracks present in the wall? A. Yes B. No
10. What is the main source of drinking water for members of your family?
11. Spring B. Dug Well C. Surface Water (River/Dam/Lake/Pond/Stream) D. Public Tap/Standpipe E. Other specify____
12. What kind of toilet facilities does your family use? A. Pit Latrine (no cement slab) B. Cemented pit latrine C. Other specify_____
13. Any possible mosquito breeding sites around your home? A. Yes B. No
14. If yes to question 20, distance of the house from mosquito breeding site?
15. <1000 m B.1000m-2000 m C.>2000 m D. Other specify_______
16. **Knowledge about malaria (Symptoms, transmission and Control measures)**
17. Have you heard about malaria? A. Yes B. No
18. If yes, where do you get the information on malaria?
19. Health services
20. Mass media (Radio and TV)
21. Religious institutions
22. Other specify_________________
23. Is malaria is transmissible A. Yes B. No
24. If your answer is “Yes” to Q26, how is malaria transmitted?
25. Contact with people with the disease
26. Contaminated food
27. Mosquito bites
28. Through flies
29. Poor personal hygiene
30. Other: ________
31. Who can contract malaria? A. Children B. Youth C. Adults D. Men E. Women F. All
32. What are the symptoms of malaria? (You can tick more than one answer)
33. Fever B. Vomiting C. Chills and Shivering D. headache E. Loss of appetite F. Joint pain
34. When do mosquitoes mostly bite? A. day B. night C. I don’t know
35. When do mosquitoes mostly bite? A. day B. night C. Any time D. I don’t know
36. . Where do mosquitoes mostly breed?
37. Stagnant water B. Running water B. Waste material C. Others specify______
38. **Attitude preventive methods of malaria**
39. Do you think that malaria is preventable and curable? A. Yes B. No C. I don’t know
40. If your reply ‘Yes’, what are the methods to prevent malaria?
41. Use of mosquito net B. Taking tablets C. House spray with insecticides D. Removing mosquito breeding site E. Other specify_______
42. Do you think malaria is a serious health problem in your area? A. Yes B. No C. I don’t know
43. What do you think the importance of insecticide treated bed nets (ITNs) in preventing malaria? Please, specify ___________________________________________________
44. **Practices of respondents towards malaria prevention and control**
45. Has anyone in your family had a fever in the last 6 months? A. Yes B. No
46. If yes, what do you do when you or your family develop malaria symptoms?
47. Self-medicate B. Visit health centre C. Drug shop/Pharmacy D. Traditional healers
48. Other specify_________
49. How long after the fever does the above-mentioned attitude take place?
50. Before 24 hours B. Before 48 hours C. After 48 hours
51. How can you prevent yourself from getting malaria?
52. Isolating infected person B. Avoiding movement or staying at home C. Smoking the house with special smoking material D. Spraying the house with chemicals E. Covering body with mosquito nets at night F. Taking chemoprophylaxis G. Avoiding pond and dams H. Cleaning bushy and swamp areas I. Specify_________________________
53. Have you ever used one of the activities you just mentioned for malaria prevention?
54. Yes B. No
55. Do you participate in any malaria control activities in the community? If yes in which way?
56. Draining logged water B. Environmental cleaning C. Educating other people D. Any other way____________________________________
57. **Mosquito Nets and Insecticide Spraying**
58. Do you have ITN in the house? A. Yes B. No
59. If yes, how many ITNs do you have in the house? A. 1 B. 2 C. 3 D. >3
60. Where do you get ITNs? A. Health centres B. Drug stores C. Local shops
61. Do you use ITNs? A. Yes B. No
62. Did you or your family use it last night? A. Yes B. NO
63. Why not use it?
64. I do not know how useful B. I do not have C. Lack of replacement schemes D. Does Heat E. Afraid of toxicity F. Other: _______________
65. Who uses the ITNs? A. Children B. Mother C. Father D. Father and Mother

E. Children and Pregnant mother F. Other family members

1. How often do you or your family sleep under ITNs? A. Regularly B. Occasionally C. During malaria season D. Other specify _____________
2. Have your home been sprayed in the last 12 months? A. Yes B. No C. Do not know
3. If yes, when was last sprayed? A >6 months B. >12 months C. Other specify___________
4. If not, why? A. Dirty wall B. causes itching C. I was not at home D. No effect E. I heard that the product does hurt people F. Others (please specify) ____________
